# Multi-branch network for double JPEG detection and localization

**DOI:** 10.1038/s41598-025-04203-0

**Published:** 2025-06-03

**Authors:** Ahmed M. Fouad, Hala H. Zayed, Ahmed Taha

**Affiliations:** 1 Faculty of Computers and Artificial Intelligence, Benha University, Benha, Egypt; 2 Faculty of Computer Science, Modern Academy, Cairo, Egypt; 3 Faculty of Engineering, Egypt University of Informatics, Cairo, Egypt

**Keywords:** Image forensics, Double JPEG detection, Double JPEG localization, Image manipulation, Convolutional neural networks, Mathematics and computing, Computer science

## Abstract

Recently, the accessibility and user-friendly nature of image editing tools have increased, allowing even inexperienced users to create and share forged images. Therefore, developing forensic methods to detect forged images is crucial. JPEG image tampering often involves recompression with a different quantization table, known as double JPEG compression. This paper proposes a multi-branch convolutional neural network and compares it with single-branch models to demonstrate its effectiveness in detecting double JPEG compression. The network consists of inter-branches, capturing statistical correlations across all Discrete Cosine Transform (DCT) frequency bands, and intra-branches, focusing on within-band correlations. By increasing feature extraction through additional intra-branches, the system enhances detection performance, particularly in complex datasets with diverse quantization tables. Features are concatenated with the image quantization table to improve robustness across varying quantization table combinations. Evaluated on the Park dataset, which includes over a million JPEG patches and 1,120 randomly assigned quantization tables, the proposed multi-branch model outperforms single-branch architectures (VGG16, DenseNet121, ResNet50) and surpasses state-of-the-art methods with a 94.15% accuracy. Furthermore, it demonstrates superior performance in localizing manipulated regions in real-world images.

## Introduction

Digital images have become one of the most important sources of information. They are widely used in various fields, such as journalism, social media, and the internet, and as evidence in courts of law, where their authenticity can significantly influence outcomes. With the widespread availability of powerful photo editing software, it has become easier for individuals to manipulate images in often undetectable ways to the human eye. This can cause a severe threat, as sharing false or altered images can lead to misinformation, reputational damage, or even societal harm. Over the years, researchers have been developing techniques to investigate the authentication of digital images^1–3^, leading to advancements in image forensics.

These manipulation detection methods are generally classified into two categories: active techniques, which embed auxiliary information like watermarks during the recording process of the image, and passive techniques, which analyze the image itself for signs of tampering, do not require any prior information about the image, such as embedded watermarks or signatures. Passive image manipulation detection techniques can be classified into five main categories. The first is pixel-based techniques^4–7^, which analyze the image at the pixel level, looking for inconsistencies such as noise patterns or pixel correlations that may indicate tampering. This category of techniques can be sensitive to noise. It can detect specific manipulations like copy-move, splicing, and retouching, but they cannot be generalized for real-world manipulation with new or various types of manipulation. The second category is Camera-based techniques^8–10^ rely on the unique characteristics of the camera that captured the image, such as sensor noise patterns or lens distortions, to detect alterations. However, their effectiveness is limited when the source of the image is unknown. Physically based techniques^11,12^ use physical world knowledge, such as lighting, shadows, and reflections, to identify inconsistencies between the scene and the image. Still, they often require precise modeling of the scene’s physical properties. Accurately determining physical inconsistencies can be challenging for complex scenes or those with unknown or non-uniform lighting conditions.

Additionally, highly skilled manipulators may be able to create realistic edits that closely mimic natural physical interactions, reducing the effectiveness of these techniques. The fourth category is geometric-based techniques^13,14^, which focus on the geometry of the objects within the image, analyzing properties like perspective, alignment, or object proportions to uncover evidence of manipulation. In some cases, perspective distortions may naturally occur due to camera angles, making distinguishing between genuine and manipulated content difficultit difficult to distinguish between genuine and manipulated content. Furthermore, advanced editing techniques that carefully maintain geometric consistency can bypass detection. Lastly, format-based techniques, which examine the structure of the image file, such as compression schemes or metadata, to identify manipulations like double compression or inconsistent quantization, are limited to specific file types and may not work effectively on formats that don’t involve heavy compression. Furthermore, sophisticated forgers can bypass these techniques by saving manipulated images in a lossless format.

However, format-based techniques can detect any forgery in any image without requiring information about the camera sensor, and they are adequate under any lighting and geometric conditions. The format-based techniques focus on the JPEG format because it is the most used image format, making the specific file’s limitation not a big deal. A study in^15^ that operated a public forensic tool for two years to determine image authenticity, analyzed 127,874 requested images. The study found that 77.95% of the images were in JPEG format. Therefore, JPEG is the most used and tamper-prone image format.

**Fig. 1:**
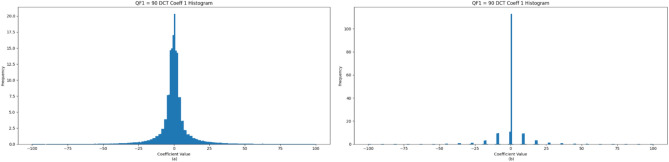
Image 8 × 8 blocks DCT histogram for the first ac coefficient (**a**) when the image is compressed with QF1 = 90 only, (**b**) when the image is double compressed with QF1 = 90 and QF2 = 60.

Double JPEG compression is a type of digital image manipulation that occurs when an image is compressed twice, often with different quantization tables or settings. When an image is saved in JPEG format, it goes through a lossy compression method designed to minimize file size by approximating the pixel values. This compression process is applied every time the image is saved, and different settings can be utilized each time. If the original image is modified and then resaved in JPEG format, the artifacts created from the initial compression may coexist with those generated by the subsequent compression. This overlapping of artifacts leads to unique patterns that suggest the image has been tampered with. As shown in Fig. 1, these artifacts appear in the histogram of the image’s DCT. Developing techniques to detect single (authentic) and double (tampered) compressed images is necessary.

## Literature review

In the literature, many studies have shown that histograms can differentiate between single- and double-compressed JPEG images. Double-compressed JPEG images are authentic images that are forged and then recompressed. Popescu et al.^16^ revealed that double JPEG compression creates distinctive periodic artifacts in the histograms of DCT coefficients, which do not appear in singly compressed images. These artifacts, seen as periodic gaps or irregularities, arise from the double quantization. They examined the histograms, mainly through Fourier transforms, and these periodic patterns can be identified, offering clear evidence of double compression. The nature of the pattern changes depending on the compression quality used in each compression step, enabling the detection of both tampering and the specific compression settings. Thus, histograms serve as a crucial tool for detecting image forgery.

Furthermore, Lin et al.^17^ presented a method to detect double JPEG by first creating histograms of the DCT coefficients for each frequency and color channel in the image. Then, they looked for periodic patterns that are caused by double compression. After identifying these patterns, a Bayesian method was used to calculate the likelihood that each image block was either tampered with or left unchanged. This approach helped the model automatically find and highlight the tampered regions, focusing on small areas with 8 × 8 pixel blocks in size. Other approaches that used handcrafted features to detect double and single JPEG compression were explored in^18–23^.

Many researchers have used the deep learning approach to detect double JPEG compression. Wang et al.^24^ analyzed histograms of DCT coefficients, which reveal distinct patterns between single and double-compressed regions. The convolutional neural network (CNN) is trained to capture these distinguishing patterns using a 9 × 11 feature matrix effectively. This matrix is constructed by extracting histograms from a specific subset of DCT coefficients, specifically the second to tenth in zigzag order. They focused on the histogram values within a defined range of bins, from −5 to 5, centered around the peak, as this range contains the most significant information about the compression characteristics. It results in better performance than handcrafted feature methods. Also, Barni et al.^25^ explored three CNN-based architectures. The first operates directly on image pixel values, allowing the CNN to learn features distinguishing between single and double compression. The second approach introduces preprocessing by applying denoising filters to images, enabling the CNN to focus on noise residuals, which improves detection in non-aligned compression scenarios. The third method computes histograms of DCT coefficients within the CNN, capturing significant traces of aligned double JPEG (DJPEG) compression. The results show that the CNN-based approach outperforms traditional methods, especially in challenging cases like non-aligned compression or when the second compression uses a lower quality factor.

Amerini et al.^26^ explored three CNN-based methods. The first is a spatial domain CNN, which operates directly on RGB image patches without preprocessing to differentiate between uncompressed, single, and double JPEG compressed regions. The second is a frequency domain CNN, which uses histograms of DCT coefficients to identify compression artifacts. Lastly, the multi-domain CNN integrates spatial and frequency domains by processing RGB patches and DCT histograms in separate CNNs and then combining their outputs to enhance detection accuracy. Moreover, Zeng et al.^27^ focused on determining the primary JPEG compression quality factor in images subjected to double compression. To improve the method’s performance, a specialized filtering layer is added at the start of the network, where filters are chosen through Fisher Linear Discriminant Analysis (F-LDA). These filters assist the network in differentiating between uncompressed, single-compressed, and double-compressed images. The architecture integrates spatial domain features learned from convolutional layers with frequency domain information derived from DCT coefficients.

Verma et al.^28^ introduced a method for classifying JPEG images according to the number of compression stages they have experienced, utilizing a CNN that operates in the DCT domain. A significant innovation of this approach is the preprocessing step, which involves directly extracting raw DCT coefficients from the JPEG bitstream and converting them into histograms for specific DCT sub-bands. These features are subsequently input into a deep CNN tailored to detect compression artifacts without being influenced by the image content. Battiato et al.^29^ introduced a CNN-based approach for estimating the first quantization matrix of double-compressed JPEG images, a crucial aspect in multimedia forensics. This method features an ensemble of CNNs trained on DCT coefficient histograms extracted from JPEG images. These CNNs are designed to operate with both DC and AC coefficients, allowing them to accommodate various patch sizes without necessitating any changes to the network architecture. Additionally, a regularization technique is employed to enhance the estimation process by taking advantage of the statistical similarities among the quantization matrix values of neighboring pixels. Gavrovska^30^ proposed a Large-Deviation Spectrum Method (LDSM), leveraging rounding and truncation (RT) errors while incorporating two additional successive recompressions to enhance classification between single JPEG (SJPEG) and double JPEG (DJPEG) images. The proposed approach demonstrates high accuracy with a minimal feature set,

Rahmati et al.^31^ employed a Convolutional Auto-Encoder (CAE) in conjunction with Convolutional Neural Networks (CNNs). The CAE functions to eliminate image content, effectively filtering out extraneous data and enabling the CNN to concentrate on identifying compression artifacts in both aligned and non-aligned DJPEG scenarios. This dual-classifier system operates on small image patches (64 × 64 pixels) to ascertain whether specific regions have experienced single or double compression. Hussain et al.^32^ approach introduces an end-to-end deep learning framework that directly processes raw DCT coefficients to distinguish between singly and doubly compressed images. The framework operates in two stages: first, an auxiliary DCT layer with sixty-four 8 × 8 DCT kernels extracts DCT coefficients dynamically rather than relying on direct extraction from the JPEG bitstream. This allows the system to analyze double-compressed images stored in spatial domain formats (e.g., PGM, TIFF, or other bitmap formats). In the second stage, a deep neural network with multiple convolutional blocks extracts high-level features for improved classification accuracy.

Several studies have focused on detecting double JPEG compression, particularly when the same quantization matrix is used. Xiaojie et al.^33^ presented a method for detecting DJPEG compression in color images, particularly in cases where the same quantization matrix is utilized for both compression stages. This approach integrated traditional feature extraction techniques with CNNs. Key features are derived from various errors resulting from color space conversion, rounding, and truncation. Manual extraction is applied to capture features related to color space conversion errors, while CNNs identify features stemming from rounding and truncation errors. The CNN architecture incorporates 1 × 1 convolutional layers and Dropout layers to mitigate the risk of overfitting, while a support vector machine (SVM) classifier is employed for the final classification.

Li et al.^34^ method that enhances detection performance by extracting highly discriminative features from error images. Their approach introduced a new error block classification scheme, categorizing blocks into stable error blocks, rounding error blocks (REBs), and truncation error blocks (TEBs). By theoretically analyzing REBs and TEBs, they identified intrinsic variables responsible for differences between singly and doubly compressed images, leading to the extraction of 25-dimensional highly discriminative features. Another notable approach exploits the component convergence during repeated JPEG compressions to distinguish between singly and doubly compressed images Niu et al.^35^. This method conducted an in-depth analysis of the rounding and truncation errors that occur during successive JPEG compressions, revealing that JPEG coefficients tend to converge after multiple recompressions. Based on this observation, the authors introduce the Backward Quantization Error (BQE) and demonstrate that the ratio of non-zero BQE is larger in singly compressed images than in doubly compressed ones. Furthermore, to fully utilize the convergence property of JPEG coefficients, a multi-threshold strategy is designed to capture statistical variations in coefficient differences between two sequential compressions. These statistical features are concatenated into a 15-dimensional feature vector, leading to an improved detection performance. However, the same quantization matrix method is specifically designed for cases where the same quantization matrix is used. In real-world scenarios, recompression often involves randomly assigned quantization tables, making detection significantly more challenging.

Liu et al.^36^ introduced an end-to-end Feature-Fusion Network (FF-Net) to detect double JPEG compression and localize image forgeries at the pixel level. FF-Net focuses on learning JPEG compression fingerprints by analyzing the high-frequency components of images. The network uses two encoders: a Spatial Rich Model (SRM) encoder to suppress semantic information and emphasize high-frequency image components and a Discrete Wavelet Transform (DWT) encoder to enhance the network’s ability to detect compression artifacts. By combining these encoders, FF-Net directly learns and detects differences in compression fingerprints between singly and doubly compressed image regions. Billa et al.^37^ introduced a CNN-based method for detecting image resizing in scenarios where Double JPEG (DJPEG) compression is present and for estimating the resizing factor applied before the second compression. A significant innovation of this approach is the inclusion of a preprocessing layer that utilizes high-pass filters to capture the residuals from the resizing process. These are valuable indicators for forensic analysis. These residuals are then fed into a CNN architecture. This method is entirely end-to-end, eliminating the necessity for handcrafted features.

All these systems share a standard limitation: they are designed to detect double JPEG compression only under specific conditions, mainly when standard quality factors are applied. However, JPEG images can be generated in real-world scenarios with various quality parameters that may not conform to these predefined standards. Different software applications and camera manufacturers often implement unique JPEG quality factors, leading to diverse compression characteristics. This variability in quality settings can significantly impact the effectiveness of the detection methods, as they may not be equipped to handle the multitude of potential combinations of quality factors that can occur in practice. To address this issue, Park et al.^15^ created a new method specifically for detecting double JPEG compression in real-world situations characterized by varying JPEG quality factors. This dataset was generated using 1,170 distinct quantization tables from 99,677 real-world JPEG images obtained through an image forensic service. Recognizing that images encountered in everyday scenarios frequently employ nonstandard quantization tables, they prioritized capturing this diversity by incorporating both standard and nonstandard JPEG quality factors. 18,946 RAW images from 15 camera models were processed to assemble the dataset. Single JPEG blocks were produced by compressing RAW blocks with randomly chosen quantization tables.

In contrast, double JPEG blocks were created by adding a random quantization table to these already compressed blocks. This dataset offers a more realistic testing environment than previous datasets, which were restricted to standard quality factors, thus making it particularly suitable for developing generalized methods for detecting double JPEG compression, then using a CNN that utilizes histogram features from each image block’s DCT coefficients, combined with the image’s quantization table, to differentiate between single and double JPEG compressed regions. Kwon et al.^38^ presented CAT-Net, a deep-learning architecture for detecting and localizing image splicing. CAT-Net is an end-to-end fully convolutional neural network that combines two parallel streams: the RGB stream and the DCT stream. The RGB stream extracts visual features from pixel data, while the DCT stream focuses on identifying compression artifacts, mainly from JPEG compression. The DCT stream is pre-trained to detect double JPEG compression, enabling it to spot spliced areas with distinct compression patterns effectively. The outputs from both streams are fused to generate a pixel-level mask highlighting the manipulated regions. The DCT stream part was trained on the same dataset by^15^. Vema et al.^39^ introduce a deep learning system for detecting forged regions in JPEG images by analyzing the relationship between histograms of quantized Discrete Cosine Transform (DCT) coefficients and their corresponding quantization step sizes. The authors propose an input representation that combines these two elements, feeding it into standard CNN architectures to differentiate between singly and doubly compressed image blocks without full decompression.

In summary, while statistical and traditional machine-learning methods have laid the foundation for double JPEG detection, recent deep-learning approaches offer significant improvements in accuracy and robustness. However, several limitations remain unaddressed in the existing literature. Most existing deep learning methods model the global DCT histogram but fail to capture both inter-frequency and intra-frequency statistical correlations, which are essential for accurately detecting double JPEG compression. We propose a multi-branch CNN, where each intra-branch models the local correlation within the histogram of a specific DCT frequency band, and the inter-branch captures the global correlation across different frequency bands, allowing for a more discriminative representation. Many prior models are computationally expensive, with high inference costs, making them unsuitable for large-scale or real-time forensic applications. We design a DenseNet-based architecture that reuses features to minimize computational redundancy. Our model achieves the lowest GFLOPs (1.119) among the compared methods while maintaining competitive detection performance. Several existing models are trained and tested under fixed JPEG quality factor (QF) settings, which limits their generalization to real-world scenarios where compression parameters vary. We evaluate our model on datasets with mixed JPEG quantization table combinations, and the results show that the proposed network maintains high detection accuracy under these more realistic conditions. The contributions of this paper are as follows: proposing a deep neural network to detect double JPEG compression by analyzing the inter-correlation across the DCT frequency histogram and the intra-correlation within each DCT frequency histogram independently, conducted experiments to optimize the network architecture and hyperparameters, ensuring the best performance for double JPEG detection, demonstrated that a multi-branch network significantly improves detection accuracy compared to single-branch architectures and can be easily integrated into existing single-branch models to enhance their performance, finally achieving superior results compared to current state-of-the-art methods in detecting and localizing image forgeries in real-life scenarios.

The rest of the paper is organized as follows. Section "The proposed architecture"presents the proposed work. Section "Experiments"describes the experimental setup and evaluation metrics used to validate our approach. The results and analysis of our experiments are also discussed. Finally, Section "Conclusion"concludes the paper, summarizing our contributions and suggesting directions for future research.

## The proposed architecture

**Fig. 2:**
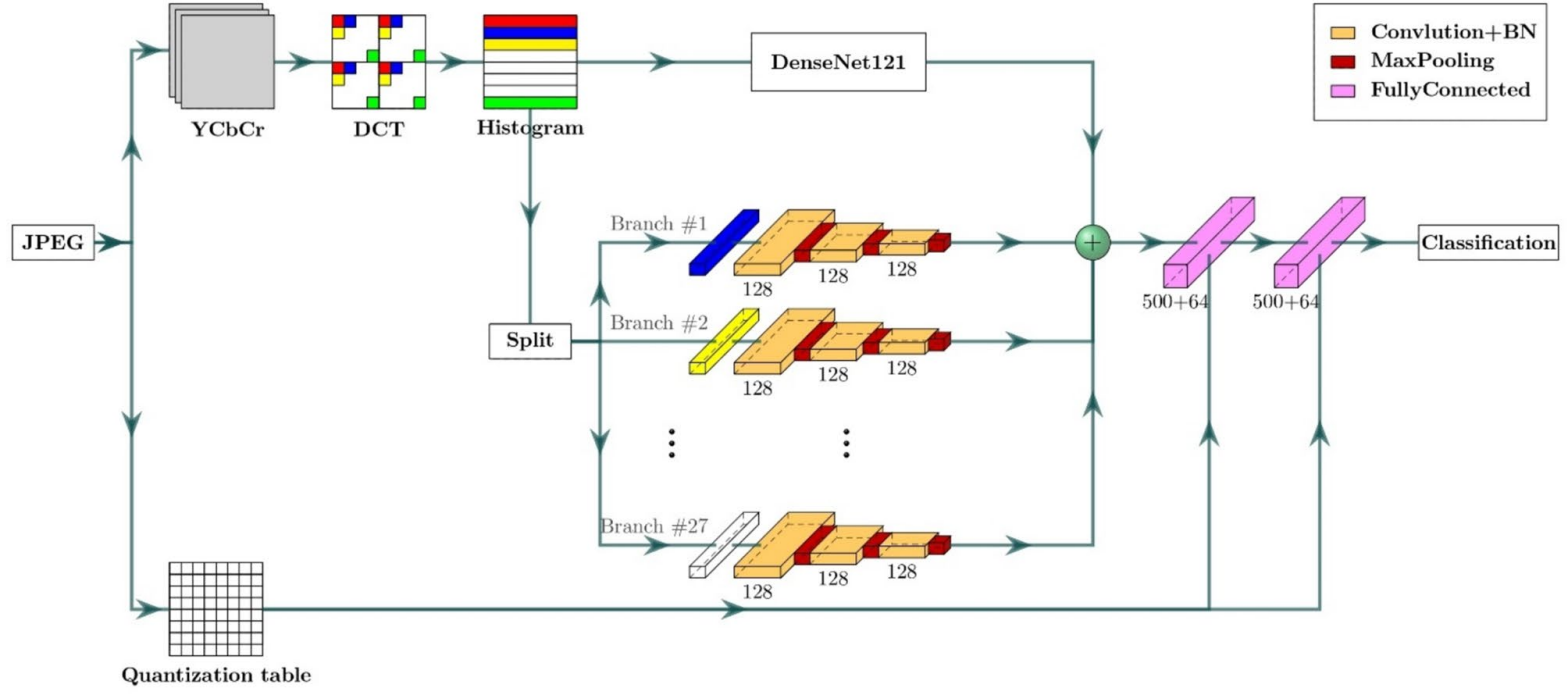
The architecture of the proposed DenseNet-based multi-branch model.

**Fig. 3:**
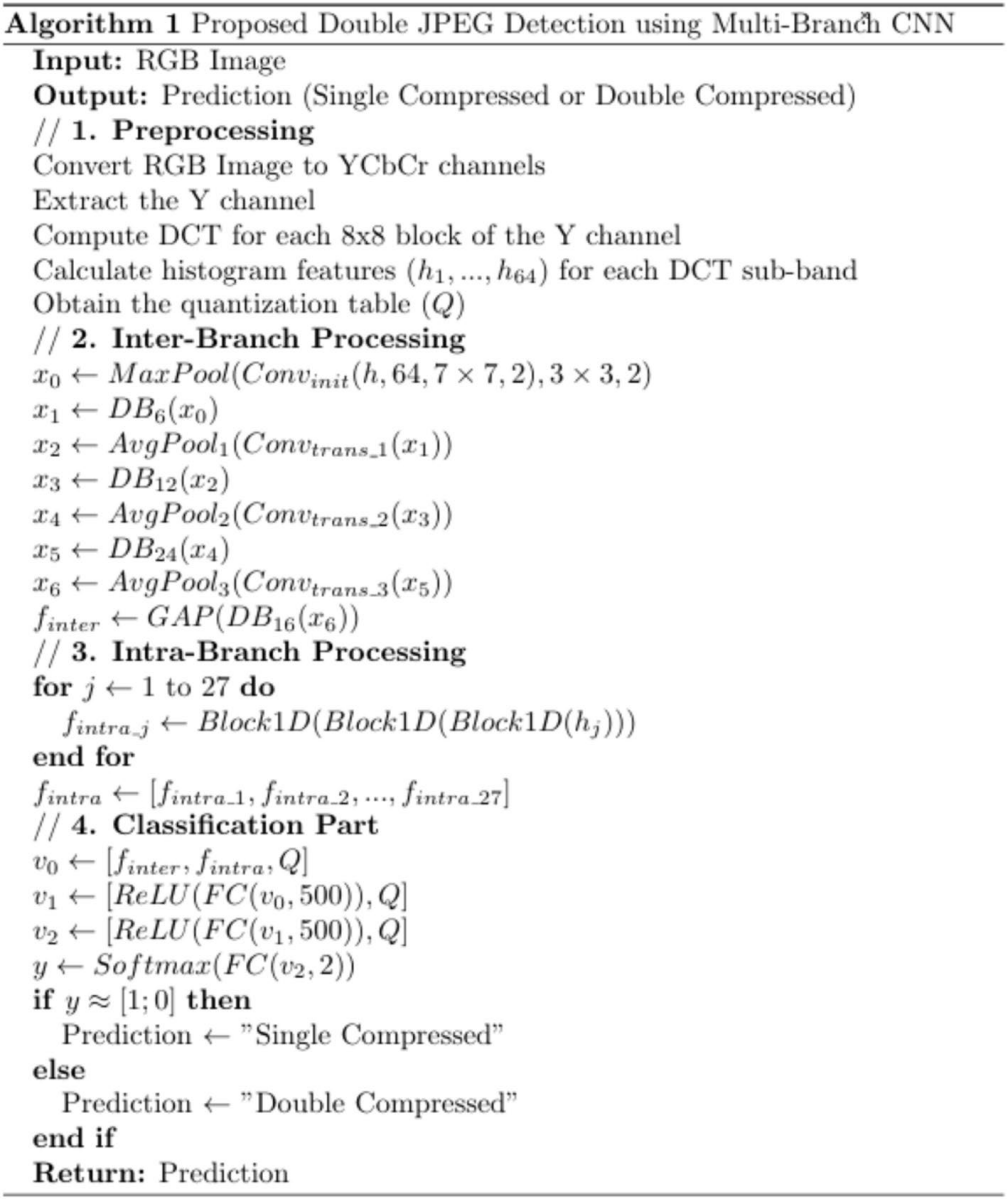
The pseudocode of the proposed DenseNet-based multi-branch model.

The main challenge is to design an architecture that can detect real-world manipulated images with any mixture of quality factors and gain the highest accuracy. This section presents the proposed double JPEG detection method using a multi-branch CNN. Our proposed method introduces a multi-branch network designed to enhance the detection of double JPEG compression. The architecture leverages multiple branches, each specializing in capturing different compression artifacts to improve detection accuracy. Figure 2 and Fig. 3 shows the overall architecture and pseudocode of the proposed multi-branch CNN. Input consists of two parts: histogram features and a quantization table. First, the RGB image is converted to YCbCr channels. For the Y channel, the Discrete Cosine Transform (DCT) is applied to each (8 × 8) non-overlapping block, as the JPEG quantization step operates on these blocks individually using the same quantization table. This means that identical coefficients are applied to the same frequency bands across all blocks, while different bands are quantized using different coefficients. We aim to capture the artifacts introduced during this quantization process, which inherently occurs at the block level. Histogram features are calculated for each sub-band to obtain an input of 64 histogram feature vectors, one for each sub-band. The quantization step in the JPEG compression process changes the statistical properties of the DCT coefficients for each (8 × 8) non-overlapping block for YCbCr channels. Input consists of histogram features with a quantization table. Each JPEG image must be stored with its quantization table to be used in the decompression process. A quantization table is used as input to the proposed network. As described before, this quantization table quantizes each (8 × 8) block. Hence, each coefficient will affect the statistical distribution of its corresponding frequency band. The quantization table is used as input to help the network distinguish if only this quantization table’s coefficients affect this image (single compressed) or if another one affects the image (double compressed).

### Architecture

**Fig. 4:**
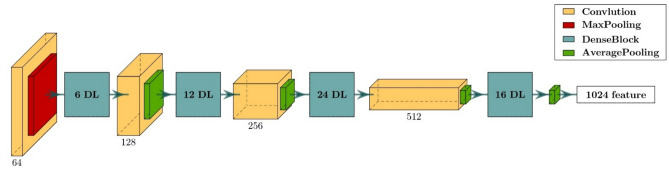
Architecture of inter-branch.

The proposed network is a multi-branch CNN, and its branches can be divided into inter-branch and intra-branch. The inter-branch is a DenseNet121^40^ network, as shown in Fig. 4. Input to this branch is the histogram features that pass through a convolution ($64$ kernels, 7 × 7, stride = 2) and max pooling (3 × 3, stride = 2) layers, then four dense blocks. Each of the first three blocks is followed by its convolution ($N$ kernels, 1 × 1, stride = 1) and average pooling (2 × 2, stride = 2) where $N = 128, 256, 512$ respectively, and the last block is followed by global average pooling (7 × 7). Each dense block includes a number of connected dense layers $DL$ where $DL = 6, 12, 24, 16$ respectively, for each dense block. Each $DL$ consists of two convolution layers ((128 kernels, 1 × 1, stride = 1), ($32$ kernels, 3 × 3, stride = 1) respectively, then a concatenation layer that concatenates the input before the convolution to the output after the convolution as described in Eq. 2.

Let ${\text{x}}_{0}$ be the input to the Dense Block. For the $\text{i}$-th Dense Layer $\left({\text{DL}}_{i}\right)$ within the block, its input ${\text{x}}_{\text{i}}$ and output ${\text{H}}_{\text{i}}$ are defined as Eq. 1:

1 $${x}_{i}=\left[{x}_{0},{H}_{1},{H}_{2},\dots ,{H}_{i-1}\right]$$

2 $${H}_{i}=D{L}_{i}\left({x}_{i}\right)=\left[{x}_{i},Con{v}_{2,i}\left(Con{v}_{1,i}\left({x}_{i}\right)\right)\right]$$

where ${\text{H}}_{\text{j}}={\text{DL}}_{\text{j}}\left({\text{x}}_{\text{j}}\right)$ is the output of the $\text{j}$-th Dense Layer, [] denotes the concatenation process, and ${\text{Conv}}_{1,\text{i}}$ and ${\text{Conv}}_{2,\text{i}}$ represent the two convolutional layers within the $\text{i}$-th Dense Layer. The output of the Dense Block after $\text{n}$ Dense Layers is Eq. 3:

3 $$D{B}_{n}=\left[{x}_{0},{H}_{1},{H}_{2},\dots ,{H}_{n}\right]$$

This branch is used to find the statistical correlation between the histogram of all 64 frequency bands.

The rest of the branches are Intra-branches. Input to each branch is the histogram of the corresponding frequency band. The input was passed through another three convolutional blocks. Equation 5 consisting of two 1D (one dimension) convolutional layers with batch normalization BN and rectified linear unit ReLU Eq. 4 (128 kernels, kernel size = 5, stride = 1). Then, each block is followed by a max pooling layer (kernel size = 2, stride = 2). Those branches are used to find statistical correlation inside the histogram for each frequency band. Only the first 27 AC frequencies in zigzag order are selected. This is the best-affected frequency sub-band in the quantization step (not very high and not very low). The quantization step has a low effect on DC frequency because it contains the image content, and the quantization step has a high impact on high frequencies, so most of them will be zero.

Let $\text{Block}1\text{D}\left(\text{x}\right)$ represent a single convolutional block defined as Eq. 4:

4 $$Block1D(x) = MaxPool(ReLU(BN(Conv1D(ReLU(BN(Conv1D(x, 128, 1))), 128, 1)))), 2, 2)$$

Then, the output ${f}_{intra\_j}$ of the intra-branch for the $\text{j}$-th frequency band with input histogram ${\text{h}}_{\text{j}}$ is given by Eq. 5:

5 $${f}_{intra\_j}=Block1D\left(Block1D\left(Block1D\left({h}_{j}\right)\right)\right)$$

The last part of the proposed model is the classification part. Features from all branches (inter and intra branches) are concatenated with quantization table coefficients to form a comprehensive feature vector.

${v}_{0}=\left[{f}_{inter},\left[{f}_{intra\_1},{f}_{intra\_2},\dots ,{f}_{intra\_27}\right],Q\right]$. This fusion process ensures that the network leverages the diverse information captured by each branch. The feature vector passes through two fully connected dense layers with units equal to 500. The output of each fully connected layer is concatenated with the quantization table coefficients as shown in Eq. 6.

6 $${v}_{1}=\left[ReLU\left(FC\left({v}_{0},500\right)\right),Q\right] {v}_{2}=\left[ReLU\left(FC\left({v}_{1},500\right)\right),Q\right]$$

Finally, the last classification layer with a SoftMax 2 × 1 vector $y = Softmax(FC(v\_2, 2))$, where $y=[1;0]$ for a single block and $y=[0;1]$ for a double block. Quantization table coefficients are concatenated with features at each step because the proposed network should work with any mixture of quality factors. The network needs to know which quantization table affects the distribution of image frequencies.

### Localization

**Fig. 5:**
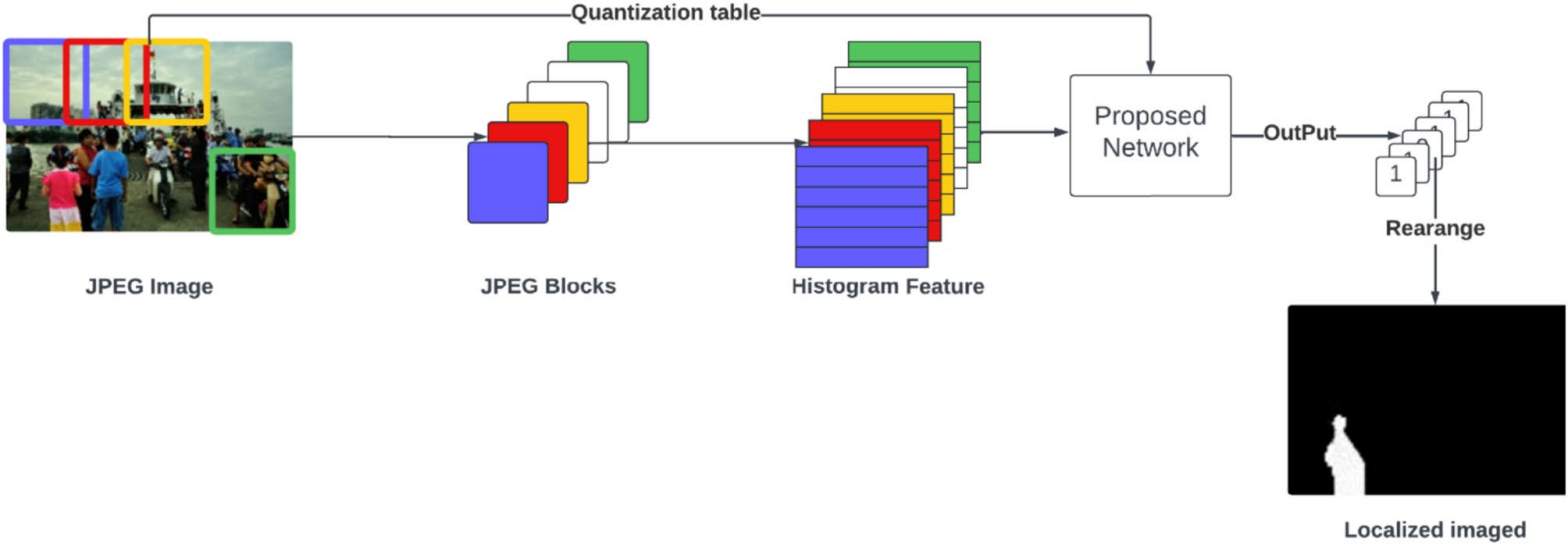
The localization process of the forged regions in a JPEG image.

A double-compressed forged image has double (authentic regions) and single (forged regions) compressed regions. So, for localizing forged regions, the image will be divided into 256 × 256 overlap blocks with a stride multiple of 8, as shown in Fig. 5. Then, each block is converted to YCbCr, and for the Y channel, DCT is calculated for each (8 × 8) non-overlapping block. Histogram features are calculated for each sub-band to obtain an input of 64 × 120 histogram features. Then, the proposed CNN is employed to calculate the probability that the block was single-compressed ${y}_{0}$. Finally, the probabilities of blocks are rearranged to create the localized image Y defined by $Y=\{y|{y}_{0}(i,j),\forall i,j\}$.

## Experiments

### Dataset and evaluation metrics

Experiments in this paper are performed on a publicly available dataset. The park dataset consists of 1,140,430 single and double compressed JPEG patches of size 256 × 256 generated using 1120 quantization tables and 18,946 RAW images captured from 15 different camera models in three different image datasets, RAISE^41^, Dresden^42^, and BOSS^43^. A total of 570,215 blocks are extracted from the RAW images. Each RAW block is compressed using a randomly chosen quantization table to generate the single JPEG blocks. Each single compressed block was recompressed using another random quantization table selected to generate double JPEG blocks.

The training process has fixed parameters of histogram bin range, train test percentage, patch size, and number of epochs with a value of [−60,60], 90%, 10%, 256 × 256, and 30, respectively. The proposed model was trained using binary cross-entropy loss and Adam optimizer^44^ with its default parameters and initial learning rate of 0.001 for the first ten epochs. Then, the learning rate is reduced by a factor of 5 for each subsequent five epochs: 0.0005, 0.0001, 0.00005, and 0.00001 for epochs 11–15, 16–20, 21–25, and 26–30, respectively.

The proposed system is evaluated and compared with other systems using metrics including accuracy, True Positive Rate (TPR), and True Negative Rate (TNR). Accuracy denotes the proportion of patches that are correctly detected as double and single compressed out of the total number of patches, and is defined by $Accuracy =(TP + TN)/(TP + FN + FP + TN)$. TPR denotes the proportion of patches correctly detected as double compressed out of the total double compressed patches, and is defined by $TPR =TP/(TP + FN)$. Similarly, TNR denotes the proportion of patches that are correctly detected as single compressed out of the total single compressed patches, and is defined by $TNR =TN/(TN + FP)$, where TP (true positive) and TN (true negative) are the number of patches correctly detected as double and single compressed, respectively. FP (false positive) and FN (false negative) are the number of patches incorrectly predicted as double and single compressed, respectively.

### Evaluation and comparison

Each branch in the proposed intra-branches consists of three convolution blocks. Each block consists of a convolution, batch normalization, and max pooling layers. Due to the long training time, the following two experiments were performed only in the first ten epochs with a fixed learning rate of 0.001. The first experiment is conducted on the intra-branches only. we conducted this experiment by varying three key parameters: the kernel size, the number of convolution layers $n \in \{\text{1,2}\}$ in convolution blocks and the number of intra-branches. Table 1 shows that increasing the number of convolutional layers from 1 to 2 (while keeping 27 branches and kernel size 128) resulted in the highest accuracy of 89.63%, with a TPR of 80.89% and a TNR of 98.36%, indicating improved learning capacity. Varying the kernel size from 50 to 256 (with 27 branches and one convolutional layer) demonstrated that a kernel size of 128 provides a strong balance between TPR and TNR, achieving up to 89.30% accuracy. Adjusting the number of branches also affected performance: while increasing to 35 slightly improved TNR, it showed a marginal decrease in TPR, resulting in a small drop in overall accuracy. These results confirm that the chosen configuration of 27 branches, kernel size 128, and 2 convolutional layers yields the best trade-off and was adopted in the final model design.

**Table 1:** Performance evaluation under varying numbers of branches, kernel sizes, and convolutional layers.

Parameters			TPR	TNR	Test accuracy
Number of branches	Kernal size	Conv layer			
27	50	1	83.54	93.98	88.54
27	128	1	80.58	**98.01**	**89.30**
27	256	1	**81.97**	95.70	88.83
20	128	1	77.78	**99.39**	88.59
27	128	1	80.58	98.01	**89.30**
35	128	1	80.26	98.22	89.24
27	128	1	80.58	98.01	89.30
27	128	2	**80.89**	**98.36**	**89.63**

Another experiment is based on the histogram of the image DCT. This experiment was conducted using the full proposed model. The experiment is to vary the number of bins in the histogram $b \in \{\text{40,80,120,160}\}$ with bin range $\{[-\text{20,20}], [-\text{40,40}], [-\text{60,60}], [-\text{80,80}]\}$ respectively. Table 2 shows that increasing the number of histogram bins to more than 120 decreases the system performance, so the value of $b = 120$ is fixed.

**Table 2:** Performance comparison with different histogram bin ranges.

Bin parameter	TPR	TNR	Test accuracy
b = 40	87.78	**98.40**	93.09
b = 80	**91.57**	95.72	93.64
b = 120	89.39	98.32	**93.86**
b = 160	89.26	97.95	93.61

A comparison between VGG16^45^, DenseNet121^40^, and ResNet50^46^ as a single branch, and when adding intra-branches is performed. All networks used the Park dataset with the same training and test batches and the same parameters. Table 3 shows that the proposed multi-branch models outperform the corresponding single-branch models, and the multi-branch DenseNet121^40^ gains the most accuracy.

**Table 3:** Single and multi-branch comparison.

Method	Single			The proposed multi		
	TPR	TNR	Test accuracy	TPR	TNR	Test accuracy
VGG16	**90.90**	94.59	92.76	90.47	**97.60**	**94.04**
DenseNet121	88.36	98.11	93.24	**90.15**	**98.14**	**94.15**
ResNet50	87.93	**97.99**	92.96	**90.59**	97.00	**93.80**

To assess the computational efficiency of our proposed model, we compared its time complexity (measured in GFLOPs) and space complexity (measured in number of parameters) with several recent state-of-the-art methods, as shown in Table 4. Our DenseNet-based multi-branch model achieves a GFLOPs score of 1.119, which is the lowest among the compared methods. The proposed model has a moderate model size of 44.8M parameters. In contrast, Kwon et al.’s model requires 23.236 GFLOPs for 114.3M parameters, while Park et al.’s and Verma et al.’s models require 3.212 GFLOPs for 16.8M parameters and 1.164 GFLOPs for 8.0M parameters, respectively. These results demonstrate that our model is significantly more efficient in terms of computational requirements, making it well-suited for applications where processing resources or runtime are limited.

**Table 4:** Time complexity comparison with state-of-the-art methods.

Method	GFLOPs	Parameters (M)
Kwon et al.^38^	23.236	114.3
Park et al.^15^	3.212	16.8
Verma et al.^39^	1.164	**8.0**
Proposed DenseNet-based model	**1.119**	44.8

**Fig. 6:**
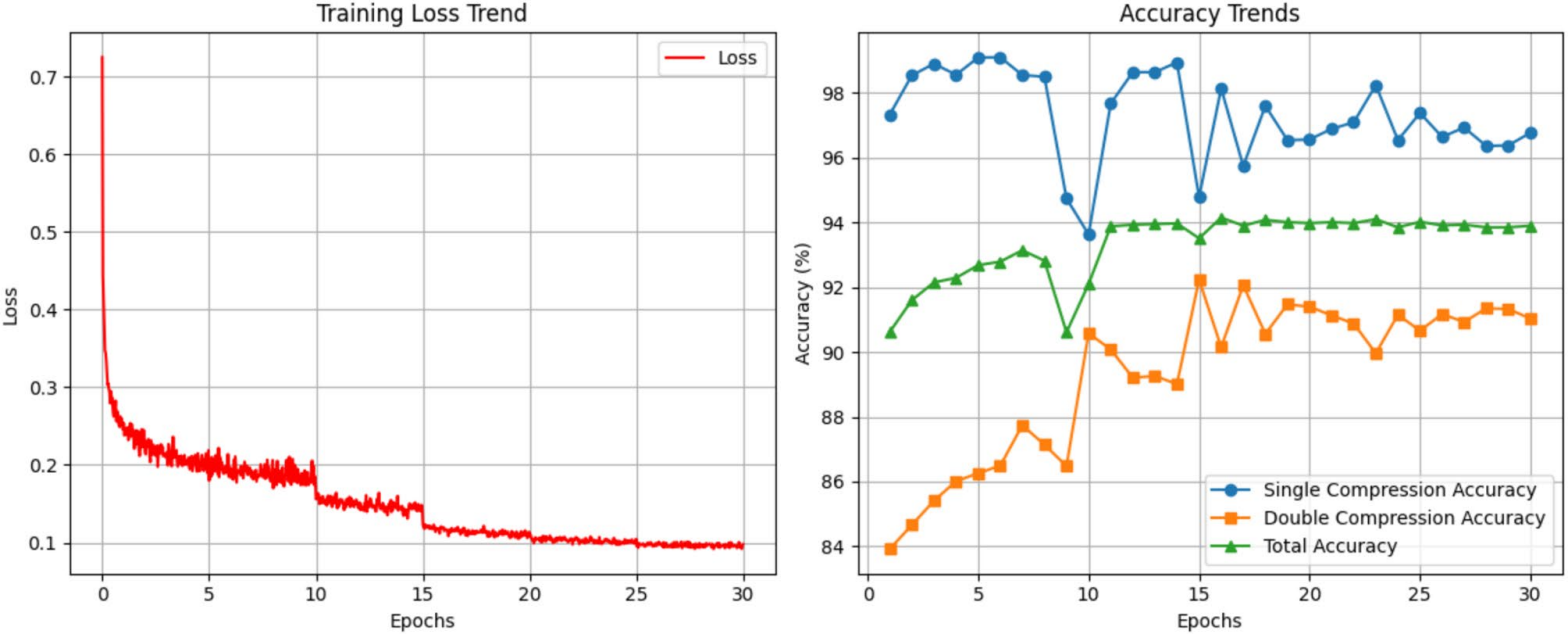
Training accuracy and loss curves of the proposed model.

To illustrate the training behavior of the proposed model, we include graphical plots of training accuracy and loss across epochs. As shown in Fig. 6, the model demonstrates stable convergence with steadily increasing accuracy and decreasing loss, indicating effective learning across a wide range of quantization configurations.

The proposed network is compared with seven networks specialized for double JPEG detection by Wang et al.^24^, Barni et al.^25^Zeng et al.^27^, Verma et al.^28^, Park et al.^15^, Kwon et al.^38^ and Verma et al.^39^. For a fair comparison, the same parameters are used, and the 90% and 10% training test is split on the same dataset for 30 epochs with a decreasing learning rate, as described before. Table 5 shows that the proposed DenseNet-based model achieves a test accuracy of 94.15%, which is highly competitive and close to the best result reported by Verma et al.^39^ (94.40%). Notably, our model achieves the highest true negative rate (TNR) at 98.14% and maintains a strong true positive rate (TPR) of 90.15%, reflecting its robustness in distinguishing both single and double JPEG compressions. In addition to its strong detection performance, our model has the lowest computational cost among all compared methods (as shown in Table 4), achieving a better balance between accuracy and efficiency, making it especially suitable for large-scale or resource-constrained forensic applications.

**Table 5:** Performance comparison with state-of-the-art methods.

Method	TPR	TNR	Test accuracy
Wang et al.^24^	67.74	78.37	73.05
Barni et al.^25^	77.47	89.43	83.47
Zeng et al.^27^	75.80	95.28	85.54
Verma et al.^28^	79.15	95.68	87.41
Park et al.^15^	90.90	94.59	92.76
Kwon et al.^38^	89.43	97.75	93.93
Verma et al.^39^	**91.87**	96.94	**94.40**
Proposed VGG-based model	90.47	97.60	94.04
Proposed DenseNet-based model	90.15	**98.14**	94.15
Proposed ResNet base model	90.59	97.00	93.80

**Fig. 7:**
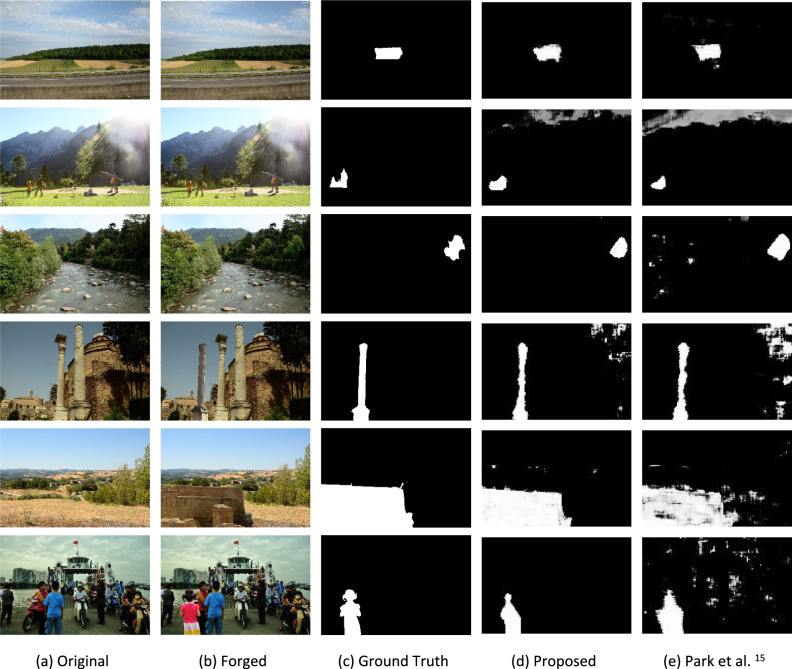
Examples of real-world manipulated images. (**a**) original images, (**b**) forged images, (**c**) the ground truth, (**d**) the result of the proposed network, and (**e**) the result of the Park et al. 15 network. The first image shows copy-move manipulation, and the second shows splicing manipulation.

The following experiment shows the localization capabilities of the proposed model on a real-world manipulated image. The images used in this experiment are created by selecting random RAW images from the RAISE^41^. A randomly selected quantization table compresses these images from the Park dataset. Further, single-compressed images are manipulated using Adobe Photoshop, and finally, manipulated images are recompressed using another random quantization table. All experiments were performed with a 256 × 256 window size and 32 strides for a fair comparison. Figure 7 shows the capability of the proposed system to detect multiple types of manipulations compared with Park et al.^15^.

## Conclusion

This paper proposes a multi-branch deep convolutional neural network to detect double JPEG compression. The branches were used to capture the compression artifact introduced in the DCT histogram of the JPEG images. Inter-branches capture the statistical correlation between the histogram of all frequency bands, and intra-branches capture the statistical correlation inside the histogram for each frequency band. Then, features from all branches are combined with respective quantization tables for classification. The proposed multi-branch model, compared with the single-branch model, is based on VGG16^45^, DenseNet121^40^, and ResNet50^46^ architectures, and the proposed multi-branch models gain more accuracy than single-branch models. The proposed system demonstrates superior accuracy compared to existing state-of-the-art methods through extensive experimentation on the benchmark Park dataset. The dataset comprises 1,140,430 single and double-compressed JPEG patches generated using 1120 standard and nonstandard quantization tables. The proposed model classifies single and double-compressed patches with an accuracy of 94.15%. The proposed model overcomes the challenge of detecting and localizing real-world handcrafted manipulated images. While the proposed model achieves strong results in detecting and localizing double JPEG compression, it inherits a known limitation of histogram-based features: difficulty in detecting double compression when the same quantization matrix is used for both compressions. In such cases, periodic artifacts are minimal or absent, making statistical differences harder to capture using histogram analysis alone. In future work, we plan to explore feature fusion strategies that combine histogram-based statistical cues with spatial-domain or frequency-domain features extracted directly from DCT coefficients or image residuals. This fusion may enhance the model’s ability to detect subtle inconsistencies in more challenging compression scenarios and improve robustness across all double JPEG configurations. Also, we aim to incorporate advanced deep-learning feature extraction techniques to improve detection accuracy, further extend the method to address other image manipulations, such as resizing and cropping, and investigate the robustness of double JPEG detection under various image distortions and noise conditions. This would require the construction or use of specialized datasets that simulate real-world manipulations, including the presence of different types of noise.

## Data Availability

The authors have used a public dataset for experimental study. This dataset is publicly available for future research and for use by other researchers https://sites.google.com/view/jspark/home/djpeg.
